# Assessing global fungal threats to humans

**DOI:** 10.1002/mlf2.12036

**Published:** 2022-09-22

**Authors:** Jianping Xu

**Affiliations:** ^1^ Department of Biology and Institute of Infectious Diseases Research McMaster University Hamilton Ontario Canada

**Keywords:** food contamination by mycotoxins, fungal allergy, fungal phytopathogens, human fungal diseases, mushroom poisoning

## Abstract

Fungi are an integral part of the earth's biosphere. They are broadly distributed in all continents and ecosystems and play a diversity of roles. Here, I review our current understanding of fungal threats to humans and describe the major factors that contribute to various threats. Among the 140,000 or so known species out of the estimated six million fungal species on Earth, about 10% directly or indirectly threaten human health and welfare. Major threats include mushroom poisoning, fungal allergies, infections of crop plants, food contamination by mycotoxins, and mycoses in humans. A growing number of factors have been identified to impact various fungal threats, including human demographics, crop distributions, anthropogenic activities, pathogen dispersals, global climate change, and/or the applications of antifungal drugs and agricultural fungicides. However, while models have been developed for analyzing various processes of individual threats and threat managements, current data are primarily descriptive and incomplete, and there are significant obstacles to integration of the diverse factors into accurate quantitative assessments of fungal threats. With increasing technological advances and concerted efforts to track the spatial and temporal data on climate and environmental variables; mycotoxins in the feed and food supply chains; fungal population dynamics in crop fields, human and animal populations, and the environment; human population demographics; and the prevalence and severities of fungal allergies and diseases, our ability to accurately assess fungal threats will improve. Such improvements should help us develop holistic strategies to manage fungal threats in the future.

## INTRODUCTION

**Figure 1:**
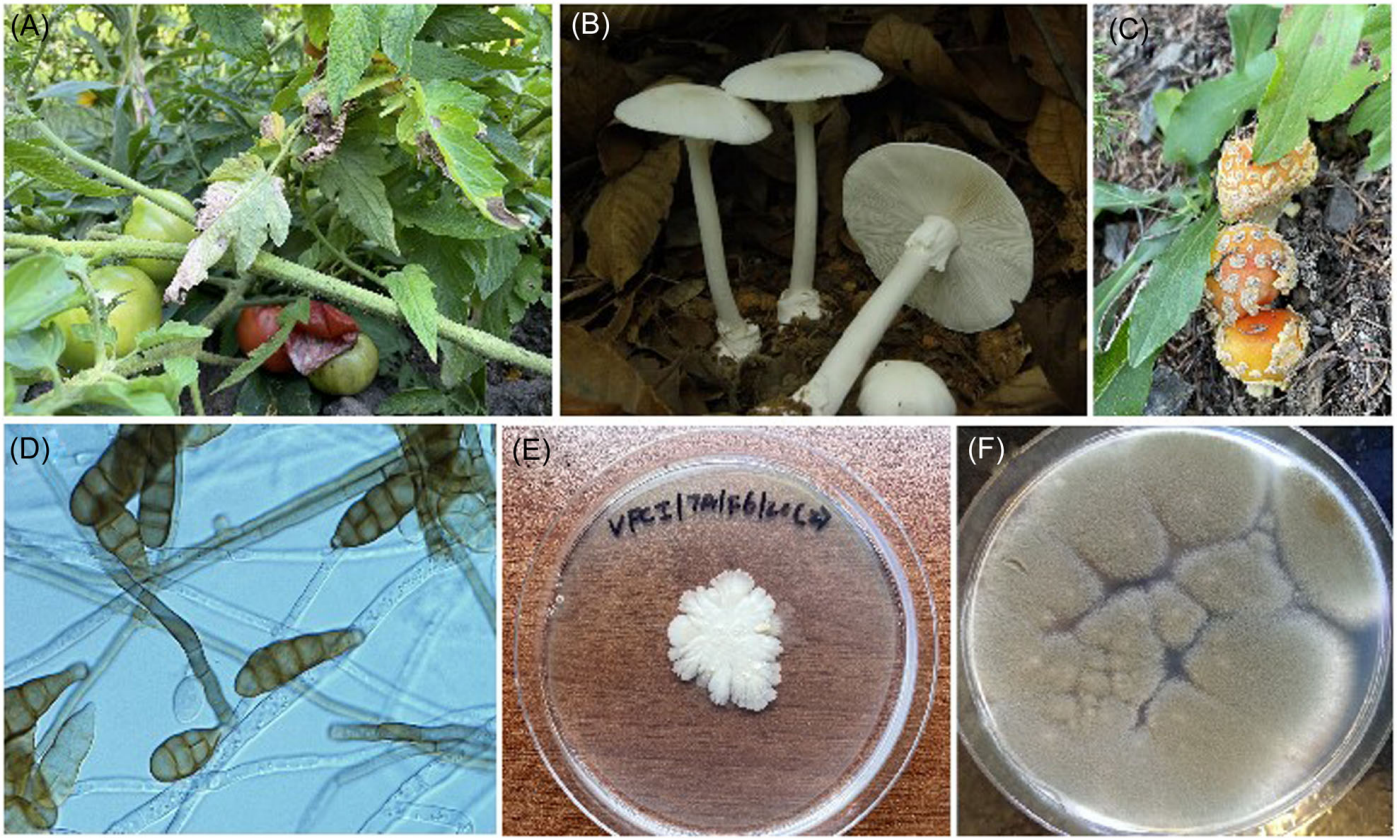
Examples of fungi threatening human health and welfare. (A) Early blight of tomato caused by *Alternaria alternata*. (B) Fruiting bodies of *Amanita exitialis*, one of the deadliest mushrooms known (photo credit: Dr. Ping Zhang). (C) Fruiting bodies of *Amanita muscaria*, an iconic poisonous mushroom in popular media. (D) Hyphae and spores of *Alternaria alternata*, a common mold in our indoor and outdoor living environments capable of causing allergies and infections in humans as well as severe diseases in dozens of crop plants (photo credit: Guangzhu Yang). (E) Colony of the yeast *Candida auris*. (F) Colony of the mold *A. fumigatus*, also common in both indoor and outdoor environments and capable of causing allergies and a diversity of invasive human infections (photo credit: Greg Korfanty). Photos of (A), (C), and (E) were taken by J. P. Xu.

Fungi are broadly distributed across the biosphere, ubiquitous in both human‐made and natural environments. They are a highly heterogeneous group of organisms in terms of their morphology, genome size and structure, metabolism, mode of reproduction, and ecological niche distribution. They play important roles in geochemical cycling, ecosystem balance, and the health of plants, animals, and humans. Everyday, throughout our lives, we encounter millions of fungal spores or filaments: through the air we breathe, the food we eat, and by contact through our skin. Among the fungi that we frequently encounter, some such as the Baker's and Brewer's yeast *Saccharomyces cerevisiae* and the white button mushroom *Agaricus bisporus* are relatively well known to the general public. However, most people are probably not familiar with yeasts in the genus *Candida* and molds in the genus *Aspergillus*. Between 30% and 60% of the general population have *Candida* yeasts in their oral cavity and each day, each of us breathe in hundreds to millions of *Aspergillus* spores^1^. In addition, each year, species in both *Candida* and *Aspergillus* genera cause millions of infections and hundreds of thousands of deaths (Figure 1). However, compared to human viral and bacterial pathogens such as SARS‐CoV, Ebola, and *Escherichia coli*, despite strong advocates from the mycological community, fungal threats have received relatively limited attention outside of the academic circle. Indeed, there are no fungi on the reportable disease list developed by the World Health Organization (WHO; https://platform.who.int/data/). The limited media attention for fungi is in stark contrast to their medical importance. For example, fungi can cause a variety of diseases in humans and many of them infect and kill more people than SARS‐CoV, Ebola, *E. coli*, and many other viral and bacterial pathogens^1^, ^2^.

Like viruses and bacteria, fungi can negatively impact humans more than just causing human infectious diseases. They can exert their detrimental effects in a variety of ways, either directly or indirectly. For example, ~8000 fungal species are known plant pathogens. Some of them cause diseases to trees, shrubs, and grasses, while others infect cereal, vegetable, and fruit crops. Together, fungal plant pathogens cause estimated 30%–40% of crop losses worth hundreds of billions of dollars each year^3^, ^4^. Similarly, many fungi can produce toxins and contaminate our food and environment. Examples of mycotoxin‐producing fungi include *Aspergillus flavus* and *Fusarium graminearum*, which produce aflatoxins and tricothecenes, respectively, causing a diversity of adverse health effects^5^. Among the fungi in our indoor and outdoor living environments, many of them such as species in the genera *Aspergillus, Cladosporium*, and *Alternaria* contain potent allergens. Overall, an estimated 4% of the global population, with up to 70% of the adults in certain geographic regions, is allergic to fungi^6^, ^7^. Furthermore, over 100 mushrooms are highly toxic to humans and these deadly mushrooms kill hundreds of people each year due to inadvertent consumptions^8^, ^9^. Though less toxic, if ingested, about 5000 additional mushroom species are known to cause a diversity of adverse effects in humans^8^, ^9^, ^10^. Together, the number of fungal species posing threats to humans, either directly or indirectly, represents about 10% of the 140,000 or so currently known fungal species.

Since the late 19th century, significant progress has been made in understanding fungal threats. These advances include identifying the diverse fungal agents of threats and the potential factors and mechanisms that contributed to their threats. This knowledge has led to the development of many mitigation strategies that are continuously being refined with new research and development. At present, almost all the research and development efforts have been devoted to past and current threats. While such efforts are necessary and have led to tremendous benefits to humans, there has been insufficient research to quantify the parameters that influence overall fungal threats and develop predictive models for fungal threats. The limited attention to fungal threat prediction may be understandable due to the large number of variables that need to be considered and the intrinsic difficulties in such endeavors. Indeed, predictions are hard and are often wrong. However, given the accelerated environmental changes, fast and diverse modes of fungal evolution, and the changing human demographics, new fungal threats will continue to emerge, and some old threats will undoubtedly re‐emerge. Thus, it is imperative that we develop quantitative approaches to fungal threat research and test potential management strategies to minimize the impact of such threats. The potential rewards for research on fungal threat quantification and predictions could be enormous for saving healthcare costs, the economy, and human lives.

Here, I review the main types of fungal threats to humans; summarize the known major drivers for past threats; identify the broad variables that need to be considered for threat quantification; discuss the recent advances and gaps of knowledge; and propose potential areas of research to advance quantitative assessments and predictions of fungal threats. While the focus is on fungal threats, I hope that this attempt will also stimulate interest for better predictions of all microbial threats.

## MUSHROOM POISONING

Picking, trading, and consumption of wild mushrooms are widespread worldwide. While the global mushroom cultivation industry was estimated at US$58.8 billion in 2021 (https://www.imarcgroup.com/mushroom-market), the monetary value of wild mushrooms remains largely unknown. Commissioned by the United Nations Food and Agriculture Organization (UN FAO), a global survey over three years (from 1999 to 2002) revealed that people in over 80 countries frequently harvest wild mushrooms as a significant source of nutrition, medicine, and income, especially for those in rural regions^11^. The survey identified a total of 2327 harvested wild mushroom species, with 2166 for food, 470 for medicine, and 181 for spiritual and other purposes. The minimal estimated value exceeded US$2 billion per year^11^, equivalent to about US$3.3 billion today. However, though the overall probability of risk is low, consumption of wild mushrooms does pose a significant risk for some people. At present, except for a few countries, data on mushroom poisoning at the global level are not known. Based on the National Poison Data System (NPDS) in the United States, from 1999 to 2016, there were 133,700 mushroom poisoning incidences (7428/year), including 47,220 that required treatments at healthcare facilities (2623/year), and 52 (2.9/year) deaths^12^. In France, from 2010 to 2017, 10,625 people (1328/year) had been poisoned by mushrooms, causing 22 deaths (2.75/year).^13^ Though differences among years were observed, there was no statistically significant temporal decline (or increase) in either the United States or France during the reported time periods^12^, ^13^. In China, the number of incidences of mushroom poisoning is not known. However, according to China CDC, during 2010–2020, mushroom poisoning resulted in 21,967 (1997/year) hospitalizations and 788 deaths (71.6/year)^8^. If China had a similar ratio of mushroom poisoning incidence to death ratio as the average between the United States and France, the estimated mushroom poisoning incidences in China would be about 109,000 per year between 2010 and 2020. Furthermore, if the rest of the global population had similar rates of wild mushroom consumption and poisoning as the average among China, France, and the United States^8^, ^12^, ^13^ (i.e., ~117,740 poisoning incidences and 77 death per year for 23.5% of the global population), the estimated numbers of global mushroom poisoning incidences and deaths would be about 501,000 and 330 per year, respectively. However, accurate estimates require monitoring and reporting at the local community level throughout all regions where wild mushrooms are consumed. Representative poisonous mushrooms that have caused deaths through ingestion in China, France, and the United States are shown in Table 1.

**Table 1:** Mushroom species reported to have caused deaths in China (2019–2021), France (2010–2017), and the United States (1999–2016).

China (2019–2021)	France (2010–2017)	United States (1999–2016)
*Amanita exitialis*	*Amanita phalloides*	*Amanita bisporigera*
*Amanita fuliginea*	*Amanita verna*	*Amanita muscaria*
*Amanita fuligineoides*	*Amanita virosa*	*Amanita pantherine*
*Amanita gymnopus*	*Galerina autumnalis*	*Amanita phalloides*
*Amanita pallidorosea*	*Galerina marginita*	*Coprinus comatus*
*Amanita pseudoporphyria*	*Gyromitra esculenta*	*Lepiota josserandii*
*Amanita rimosa*	*Helvella crispa*	
*Amanita rufoferruginea*	*Lepiota brunneoincarnata*	
*Amanita subjunquillea*	*Lepiota helveola*	
*Amanita subpallidorosea*	*Lepiota josserandii*	
*Chlorophyllum molybdites*		
*Galerina sulciceps*		
*Lepiota brunneoincarnata*		
*Paxillus involutus*		
*Russula subnigricans*		

Data extracted from references ^8^, ^12^, ^13^.

While certain selected jurisdictions track the incidences, hospitalizations, and/or deaths due to mushroom poisoning, the quantitative contributions of factors to various forms of mushroom poisoning are often not known. To help analyze the potential factors that may contribute to mushroom poisoning, let us start with the following simplified approximation:

(1) $$N_{\text{MP}}=N_{T}\cdot F_{E}\cdot N_{P}\cdot F_{C}\cdot F_{A}$$

where *N_MP_* represents the number of people experiencing mushroom poisoning; *N_T_* is the total number of people in the population under consideration; *F_E_* is the percentage of the population consuming wild mushrooms as part of their diet; *N_P_* is the number of wild poisonous mushroom species in the surrounding environment and markets; *F_C_* is the percentage of wild poisonous mushrooms not correctly identified by pickers/consumers as being poisonous; *F_A_* is the fraction of the consumed wild mushrooms that are poisonous and with the consumption level exceeding a threshold quantity to cause poisoning symptoms, including hospitalizations and death.

At present, while the population sizes *N_T_* of most communities are known, the quantitative data on *F_E_*, *N_P_*, *F_C_*, and *F_A_* are not known but can be obtained through systematic surveys. It is likely that the parameters will vary significantly among geographic regions. For example, population density is not evenly distributed among countries and regions and people living in urban areas in general are less likely than those living in rural and forested areas to pick and consume wild mushrooms on a regular basis^11^. Second, poisonous mushrooms are not evenly distributed among geographic regions and ecological niches^8^, ^9^, ^10^, ^12^, ^13^. Third, mushroom recognition skills can differ among people, including among both pickers and consumers. Finally, how the collected mushrooms are prepared for food and the quantity consumed can also vary and have a significant influence on the health outcome of consumers.

Reduction in each of the above terms could all lead to reduced incidences of mushroom poisoning. Among the strategies, the most effective one to reduce mushroom poisoning is likely through targeted education of mushroom pickers and consumers in regions with concentrated cases of mushroom poisoning. Such campaigns can minimize *F_C_*, the percentage of wild poisonous mushrooms not correctly identified by pickers/consumers as being poisonous. Indeed, through extensive social media campaign and public health education, the number of deaths due to mushroom poisoning in China has reduced significantly over the last several years. Specifically, while the mean number of death due to mushroom poisoning from 2010 to 2020 was 71.9 per year, the numbers from the last 3 years were 22, 25, and 20 for 2019, 2020, and 2021, respectively^14^. The educational success in China is further reflected in the per capita death rate comparisons with France due to mushroom poisoning. Specifically, while the average number of per capita death due to mushroom poisoning in China between 2010 and 2020 (71.9/year) was >25 times higher than that in France between 2010 and 2017 (2.75/year), the number in China from 2019 to 2021 was only about half of that in France between 2010 and 2017 (Data in France between 2018 and 2021 are not available for comparison). It should be noted that, even though significant progress has been made in China, its per capita annual death from mushroom poisoning from 2019 to 2021 was still twice the per capita average in the United States from 1999 to 2016 (Data in the United States between 2017 and 2021 are not available for comparison).

For several reasons, even with enhanced education, as long as wild mushrooms are picked for consumption, the threat of mushroom poisoning, including death, will most likely persist. First, among the 100 or so highly poisonous mushrooms known so far, many have morphological features very similar to those that are edible. For novice mushroom pickers and even some experienced pickers, the poisonous mushrooms can be easily mistaken as being edible. Second, most of the highly poisonous mushrooms can cause severe physiological damages even when a small amount (e.g., part of a fruiting body) is consumed. Third, an increasing proportion of the traditionally considered “safe to eat” mushrooms are being reported to cause health problems in variable subsets of the consumer population. For example, the 2004 UN FAO report listed 27 species with conflicting reports of edibility among people from either the same or different regions^11^. In a 2019 publication of the Chinese macrofungi resource diversity where the edibility of 1662 species was reviewed, 480 were considered poisonous mushrooms, including 193 that had been reported as edible and/or medicinal species^9^. Indeed, the list of poisonous mushroom species is growing. In 2021 alone, China CDC identified 15 new poisonous mushrooms in China, including three species in three different genera that were previously reported as edible and/or medicinal: *Coprinellus micaceus* (a traditional medicinal mushroom), *Coprinopsis atramentaria* (a common wild edible mushroom), and *Coprinus comatus* (a widely cultivated mushroom).^14^ Interestingly, all three species produce coprine, which is especially abundant in mature fruiting bodies. When consumed with alcohol, coprine can cause disulfiram‐like mushroom poisoning^14^. Even the most widely cultivated mushroom *A. bisporus* was recently reported to cause generalized urticaria, abdominal pain, and vomiting 15 min in a teenage girl in Spain after she ate mushroom lasagna^15^. Fourth, with climate change and anthropogenic activities, the geographic ranges of poisonous mushrooms will likely experience accelerated expansion to new territories^16^, increasing the likelihood of poisonous mushrooms not being recognized and mistakenly being picked and consumed.

Recent genomic and metabolic analyses on the distributions of mushroom toxin‐producing genes and mushroom toxins among evolutionarily highly divergent taxa revealed further complications about threats posed by poisonous mushrooms^17^, ^18^. For example, one of the most potent groups of mushroom toxins, the amanitins (a group of cyclopeptides), are produced by mushrooms broadly but not universally distributed among three genera/families of mushrooms: *Amanita* (Amanitaceae) (Figure 1), *Galerina* (Hymenogastraceae), and *Lepiota* (Agaricaceae)^9^, ^13^, ^17^. In addition, variable numbers of species in these genera have the MSDIN gene family, which could be catalyzed by prolyl oligopeptidases (POP) and biosynthesize many other cyclopeptides (and some of them are also toxic). The phylogeny of one of the *POP* genes, *POPB*, showed gene genealogical relationships different from the species and genera relationships among *Amanita*, *Galerina*, and *Lepiota*, a result consistent with horizontal gene transfer among these three genera^17^. Similarly, the production of psychedelics (psilocybin and psilocin) has been reported in a polyphyletic assemblage of species in at least nine mushroom genera: *Conocybe, Galerina, Gymnopilus, Inocybe, Massospora, Panaeolus, Pholiotina, Pluteus*, and *Psilocybe* ^18^. Interestingly, two species in the genus *Massospora, M. platypediae* and *M. levispora*, lack the typical *Psi* genes involved in psilocybin biosynthesis found in other species but can still produce psilocybin^19^, ^20^. These results suggest that mushroom toxins may originate de novo spontaneously and/or that toxin‐producing genes can be horizontally transferred among divergent fungi. Indeed, the expanding geographic distributions of poisonous mushrooms due to climate change and human activities will increase the likelihood of interactions among divergent fungi and mushrooms (e.g., between edible ones and poisonous ones), potentially accelerating horizontal transfers of mushroom toxin‐producing genes among species.

While the rates of changes in the above features are largely unknown, a summary of how these factors could potentially contribute to changes in mushroom poisoning may be approximated below.

(2) $$N_{MP+T}=N_{\text{MP}}\cdot {\left(1+r_{\text{nt}}\right)}^{n}\cdot {\left(1+r_{\text{np}}\right)}^{n}\cdot {\left(1+r_{\text{fc}}\right)}^{n}\cdot {\left(1+r_{\text{fe}}\right)}^{n}\cdot {\left(1+r_{\text{fa}}\right)}^{n}\cdot {\left(1+r_{\text{mp}}\right)}^{n}$$

where *N_MP+T_* represents the number of people who experience mushroom poisoning after time *T*; *r_nt_* is the rate of change in the total number of people in the population under consideration per unit time (e.g., per year); *r_np_* is the rate of change in the number of wild poisonous mushroom species in the surrounding environment and the markets per unit time; *r_fc_* is the rate of change in the ratio of poisonous mushrooms not correctly identified by pickers/consumers as being poisonous per unit time; *r_fe_* is the rate of change in the fraction of the population consuming wild mushrooms as part of their diet per unit time; *r_fa_* is the rate of change in the fraction of the consumed wild mushrooms being poisonous and with the consumed level exceeding a threshold quantity to cause poisoning symptoms, including death per unit time; *r_mp_* is the rate of origination of new poisonous mushrooms per unit time; and *n* is the number of time units (e.g., if per unit time was 1 year, a decade would be equal to 10 time units). Similar to the terms in Equation (1), the rates of changes in Equation (2) could be estimated based on long‐term monitoring and/or comparative evolution genomic studies. Efforts to track picking and consumption patterns of wild mushrooms could help identify the key factors contributing to incidences of mushroom poisoning. Such data will be essential to design targeted programs to minimize the detrimental effects of wild mushroom consumptions while maintaining the benefits of wild mushroom harvesting.

Overall, the threat of mushroom poisoning is related to multiple interacting factors, including cultural, economic, historical, social, educational, geographic, environmental, and biological factors. Wild mushrooms will continue to be a significant source of nutrition and income for many people. However, coupled with increasing educational efforts (e.g., organizing training sessions at the beginning of each mushroom fruiting season for all potential pickers in each community), rigorous monitoring of the influences of environmental change, anthropogenic activities, and biological evolution on the distributions of poisonous mushrooms and mushroom toxins should help minimize mushroom poisoning while maximize the enjoyment of wild edible mushrooms in the future.

## FUNGAL ALLERGENS

Allergies are our body's abnormal physiological reaction to a normally harmless substance. Allergy symptoms range from mild, such as watery/red eyes, runny nose, itchiness, and rash or hives, to life‐threatening and can impact a range of organs, including the skin, the eyes, the upper and lower respiratory tract, the mouth, and the intestinal tract. Depending on the affected organ, allergic symptoms may manifest as insect venom allergy, immunoglobin E (IgE)‐associated atopic dermatitis, allergic rhinitis, allergic asthma, food allergy, and so forth. Allergies can be caused by a diversity of proteins originated from plants (e.g., pollen and peanuts), animals (e.g., cat dander and insect stings), and fungi (e.g., mold spores)^21^, ^22^. The diverse allergens have similar mechanisms of action. Specifically, upon exposure to an allergen, the immune system typically produces IgE (for Type I hypersensitivity, the most common type) and releases chemicals like histamine into the bloodstream to help the body eliminate allergens, causing the diverse allergy symptoms. However, other immunoglobins (IgG and IgM) and T cells can also be involved in our body's hypersensitive responses (i.e., Type II, Type III, and Type IV hypersensitivity). Generally, the stronger the immune system reaction to an allergen, the more severe the symptoms.

Reports of symptoms like allergies date back >3000 years ago from China, Egypt, and Rome^21^. However, most early reports were not well documented until about 1869, when Dr. Charles H. Blackley demonstrated that pollen was the cause of his hay fever^23^. It is estimated that more than 30% of the population in industrialized countries suffers from allergies^22^. At present, the exact prevalence of the general population with allergies to various fungi is not known. Based on a large skin prick test in Austria over 20 years ago on a cohort of 4962 subjects with respiratory illness conducted with commercial fungal extracts from seven common fungi, *Alternaria alternata, Aspergillus fumigatus, Candida albicans, Cladosporium herbarum, Penicillium notatum, S. cerevisiae*, and *Trichophyton mentagrophytes*, an overall prevalence of sensitization of 19.1% to these fungi was found^6^ (It should be noted that in this study, not all individuals were tested for all seven fungal extracts). The highest sensitization rate was with *A. alternata* extract (12.6%), followed by that of *C. albicans* (8.5%), *C. herbarum* (2.5%), *A. fumigatus* (2.4%), *T. mentagrophytes* (1.9%), *P. notatum* (1.5%), and *S. cerevisiae* (1.4%). About 10% of the surveyed individuals were sensitized to two of the seven fungal extracts, and 12.4% were sensitized to three or more species^6^. Interestingly, those sensitized to *A. alternata* extract were much younger (mean age 16.5 years) than the groups sensitized to *C. albicans* (29.6 years) and *T. metagrophytes* (28.5 years). Three of the seven tested species, *A. alternata, A. fumigatus*, and *C. albicans*, are among the most prevalent fungi in our living environments (Figure 1). A more recent estimate suggested that globally, 30%–70% of adults may have been sensitized to fungal allergens^24^.

A general approximation to summarize how various broad factors could impact the number of people suffering from fungal allergies is as follows:

(3) $$N_{\text{FA}}=N_{T}\cdot N_{A}\cdot F_{E}\cdot F_{A}$$

where *N_FA_* represents the number of people suffering from fungal allergies; *N_T_* is the total number of people in the population under consideration; *N_A_* is the number of known fungal allergens around our living and working environments; *F_E_* is the fraction of the population exposed to fungal allergens; and *F_A_* is the fraction of those exposed to fungal allergens experiencing allergic reactions. Except for some newborns kept in incubators, virtually everyone in the world is exposed to at least one fungal allergen every day: in the air we breathe, food we eat, or contact through our skins. Due to the ubiquitous distributions of fungi, our daily exposure to fungal allergens is unlikely to change soon.

Several factors will contribute to changes in the number of people who suffer from fungal allergies. First, climate change will lead to more flooding and high moisture in the air, causing more fungal growth and more fungal spores to be released into our living space, increasing our exposures to these known fungal allergens^25^, ^26^, ^27^. For example, between 2002 and 2019, there was a notable extension of the time frame, at 3.57 days per year, where a significant number of mold spores were detected in the outdoor air samples in the San Francisco Bay area in the United States^25^. A recent systematic review of 74 studies from Europe revealed that the peak concentrations of spores in two fungal genera, *Alternaria* and *Cladosporium*, exceeded clinical thresholds in nearly all locations across Europe, with median peak daily concentrations of 665 and 18,827 spores per cubic meter of air, respectively, for these two genera^26^. With typical adults inhaling 14 (female) to 18 (male) cubic meters of air per day, we are exposed up to hundreds of thousands of fungal spores every single day through our breathing. Similar to those found in San Francisco, meteorological variables such as temperature, precipitation, and relative humidity were all found to contribute to variations in spore concentrations in Europe^26^. In addition, people living closer to agricultural fields and coastal areas are generally exposed to higher concentrations of fungal spores. Second, because of the changing climate and environments, the distribution range of more fungi is expanding; some of these fungi may become new sources of allergens and allergies to local residents^27^. Third, previous analyses have shown that people in industrialized countries and urbanized populations were about twice as likely as those in developing countries to suffer from allergies, including fungal allergies^28^. At present, while various hypotheses have been proposed, the mechanisms for such differences among economic classes and geographic regions remain unresolved^29^. However, if such a trend was to continue, with increasing industrialization, a greater proportion of the global population will become susceptible to fungal allergens. In 2022, the total global population has been estimated at 7,866,952,023, with 1,568,433,512 being in developed countries (defined here as those with human development index >0.8) and the remaining 6,298,518,511 in developing countries (https://worldpopulationreview.com/country-rankings/developed-countries). By 2050, the global population is expected to reach over 9 billion and more countries will likely enter the developed state. If we assume the same prevalence of fungal allergies in the newly developed economies as in those from before 2022, an estimated 3 billion people in the world may suffer from regular fungal allergies throughout the year. However, many other factors could influence the predicted number of fungal allergies. Following Equation (3) and the above discussion, a generalized approximation about the potential change in the number of people experiencing fungal allergies in a community may be expressed as

(4) $$N_{\text{FA}+T}=N_{\text{FA}}\cdot {\left(1+r_{c}\right)}^{n}\cdot {\left(1+r_{a}\right)}^{n}\cdot {\left(1+r_{p}\right)}^{n}\cdot {\left(1+r_{f}\right)}^{n}$$

where *N_FA + T_* is the number of people who suffer from fungal allergies after time *T*; *r_c_* is the rate of population size change over a specific time unit (e.g., a year); *r_a_* is the rate of change in the number of people being exposed to fungal allergen over a specific time unit; *r_p_* is the rate of change in the number of exposed people being allergic over a specific time unit; *r_f_* is the rate of change in the number of fungal allergens in the environment over a specific time unit; and *n* is the number of time units (e.g., number of years). While factors that increase human fungal allergies were mentioned above, there are also factors that can contribute to decreasing fungal allergies. For example, efforts to improve air quality have been found to reduce fungal allergens in the air. Between 2001 and 2021, in the Novosibirsk region of Russia, the mean observed cultured microorganisms (including fungi) in the air decreased by 4.2%–6.6% per year among three sampled sites^30^.

At present, the exact rates of changes in *r_a_*, *r_p_*, and *r_f_* and how they may interact to influence the incidences of fungal allergies are unknown in any geographic region. Long‐term monitoring programs need to be established in representative jurisdictions and ecological niches to track the abundance of fungi and fungal allergens, record relevant ecological factors, and monitor the prevalence of fungal allergies to help establish empirical parameters and models for predicting future changes and designing management strategies. Because there may be significant differences in both the total fungal spore load and the fungal species composition in the atmosphere among seasons within the same year in each geographic region^25^, ^26^, ^27^, ^28^, ^29^, ^30^, ^31^, such monitoring programs need to include temporal samples from different months/seasons within each year.

The World Health Organization and the International Union of Immunological Societies (WHO/IUIS) Allergen Nomenclature Subcommittee have formally recognized 120 fungal allergens (Table 2; www.allergen.org; accessed June 22, 2022). These registered fungal allergens were from 32 species, with the highest number from *A. fumigatus* (30 allergens) and the second highest from *A. alternata* (12 allergens) (Table 2). Both *A. fumigatus* and *A. alternata* are ubiquitously distributed in both our indoor and outdoor living environments. In this database, a total of 1060 allergens have been registered, with 442 from animals, 498 from plants, and 120 (~11%) from fungi. However, these numbers are likely vastly underestimated. Based on structural motifs of known allergens at that time, a 2003 bioinformatics analysis of 135,850 protein entries deposited in the Swiss‐Prot database predicted that 4768 (about 3.5%) contained potential allergen structures^32^. If a similar percentage of 3.5% were potentially allergenic, the current database of 14,582,961 protein entries in the UniProt database, with 180,398 being from fungi (https://www.uniprot.org/taxonomy/4751; accessed June 22, 2022), would suggest that there may be over 6000 fungal allergens among the deposited proteins. However, further confirmation is needed about the number and nature of fungal allergens within and outside of the current databases. Once confirmed, these allergens could be developed for fast and effective allergen diagnosis and for the design of targeted desensitization programs in the future to reduce the size of the susceptible host population^33^.

**Table 2:** Distribution of confirmed fungal allergens among species.

Fungi	Route of exposure	Number of allergens
Ascomycota		
*Alternaria alternata*	Airway	12
*Aspergillus flavus*	Airway	1
*Aspergillus fumigatus*	Airway	30
*Aspergillus niger*	Airway	3
*Aspergillus oryzae*	Airway	2
*Aspergillus terreus*	Airway	1
*Aspergillus versicolor*	Airway	1
*Candida albicans*	Airway	2
*Candida boidinii*	Airway	1
*Cladosporium cladosporioides*	Airway	2
*Cladosporium herbarum*	Airway	8
*Curvularia lunata*	Airway	4
*Epicoccum purpurascens*	Airway	1
*Fusarium culmorum*	Airway	2
*Fusarium proliferatum*	Airway	2
*Fusarium brevicompactum*	Airway	2
*Penicillium chrysogenus*	Airway	6
*Penicillium citrinum*	Airway	7
*Penicillium crustosum*	Airway	1
*Penicillium oxalicum*	Airway	1
*Stachybotrys chartarum*	Airway	1
*Trichophyton rubrum*	Contact	2
*Trichophyton tonsurans*	Contact	2
*Ulocladium chrtarum*	Airway	1
Basidiomycota		
*Coprinus comatus*	Airway	5
*Malassezia furfur*	Contact	3
*Malassezia sympodialis*	Contact	10
*Psilocybe cubensis*	Airway	2
*Rhodotorula mucilaginosa*	Airway	2
*Schizophyllum commune*	Airway	1
Zygomycota		
*Rhizopus oryzae*	Airway	2

Original data were obtained from www.allergen.org on June 22, 2022.

## FUNGAL THREATS TO THE HUMAN FOOD CHAIN

Humans are omnivores. However, depending on food availability and personal preference, a range of food supply chains exist that can differ widely among individuals within most societies and countries. Overall, we consume a diversity of primary producers (autotrophs, mostly plants and some algae), saprophytes (e.g., mushrooms), and organisms at various intermediate trophic levels above primary producers. Those at intermediate trophic levels may include primary consumers such as herbivores (e.g., cows and sheep) to omnivores (e.g., wild chicken, wild boars, and many fish) and carnivores (e.g., some marine predators and wild game animals).

Among the macronutrients that we consume, almost all our carbohydrates are obtained directly from primary producers, while proteins and fatty acids may be from a diversity of trophic levels. Different from most other omnivores, humans tend to process food after harvesting to modify the flavor, nutritional value, and/or shelf‐life through baking, brewing, pickling, preserving, and cooking. For all the consumed organisms and in each of the processing steps, fungi can have detrimental effects on both the quality and the quantity of food products. In this section, I describe and discuss major fungal threats to two of the components: the health of crop plants and the quality of their postharvest products (next section).

**Figure 2:**
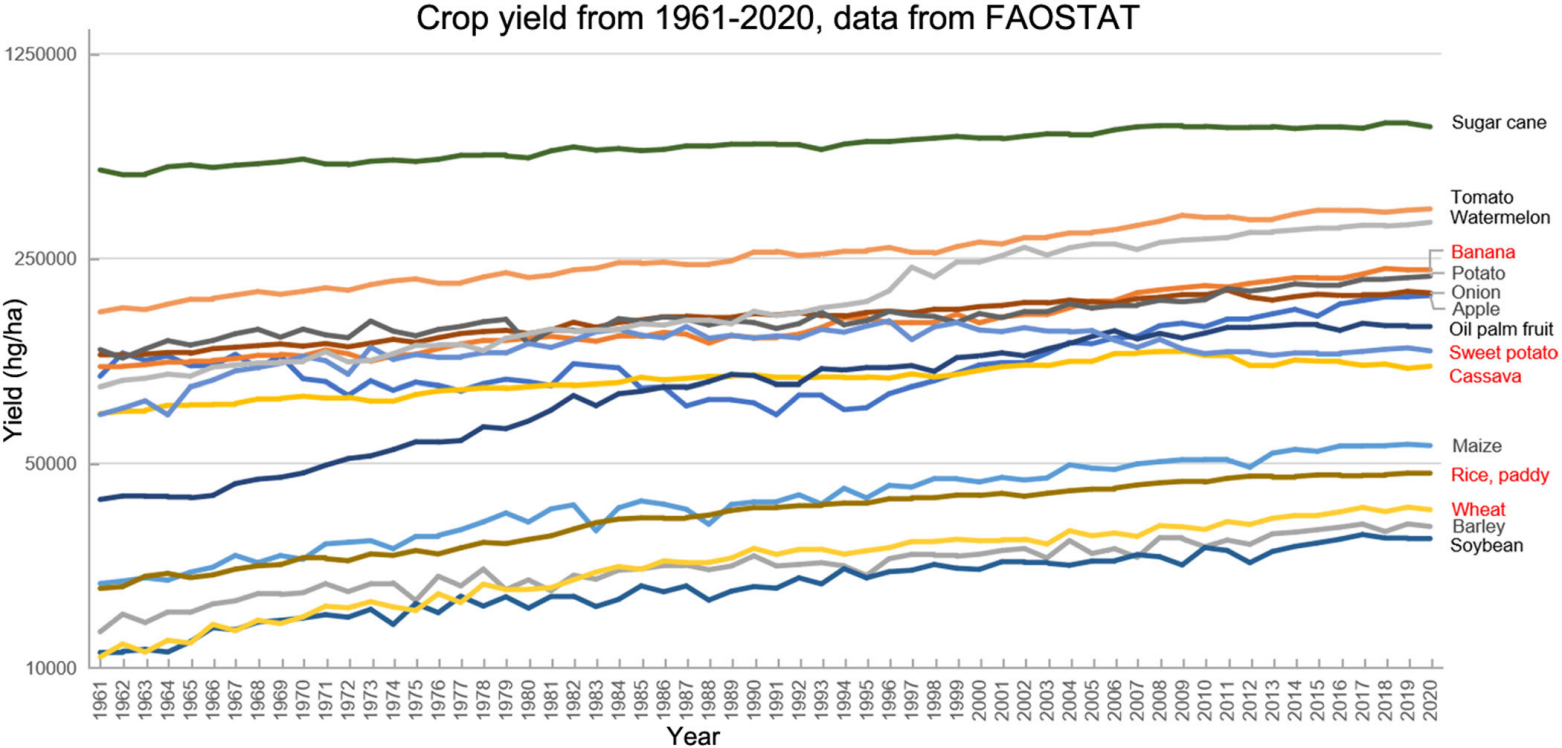
Changes in crop yield (hg/ha) for 15 food plants from 1961 to 2020. These 15 food plants had the highest tonnage in the year 2020 based on the FAOSTAT record (fao.org) from where the original data for this graph were obtained. Crop names highlighted in red are those discussed in the main text.

The UN FAO estimated that ~250,000 plant species/varieties are available for agriculture. Among these, ~7000 (representing <3%) are in use today (fao.org). However, our food supply primarily depends on ~150 plant species, with wheat, rice, and maize providing about 50% of the world's plant‐derived calories that we consume^34^. The top 15 food plants based on the total production volume in 2020 are shown in Table 3. Over the 60 years from 1961 to 2020, the global yields (hg/ha) for all top 15 food plants increased significantly, with most of them showing a general trend of continued increase (Figure 2). The increased yields were due to several factors, including development of new and more productive cultivars, increased application of fertilizers, improved cultivation techniques and crop management, and enhanced control of diseases and pests. However, for many crops, the annual rates of yield growth are declining (Figure 2). For example, the average growth rate of rice yield was 2.98% per year in 1981–1990, 0.82% per year in 1991–2000, 0.75% per year in 2001–2010, and only 0.31% per year in 2011–2020 (Figure 2). In addition, for each crop, the overall increase was punctuated by sporadic drops, with sweet potato and cassava showing notable and almost continuous declines since 1998 and 2009, respectively (Figure 2). With the expected increase in global human population size but decreasing arable land, it is essential that we increase or at least maintain the yields of crop plants to ensure global food security.

**Table 3:** Top 15 global food crops based on production tonnage in 2020 and the numbers and examples of known fungal diseases and disease agents associated with each crop.

Crop	Global production (tons)	Number of known fungal diseases/disease agents	Representative fungal diseases (representative disease agents for each disease)
Sugarcane	1,869,715,086	40/57	Red rot (*Colletotrichum falcatum*); smut (*Ustilago scitaminea*); wilt (*Fusarium sacchari*); sett rot (*Ceratocystis paradoxa*)
Maize	1,162,352,997	100/165	Anthracnose (*Colletotrichum graminicola*); charcoal rot (*Macrophomina phaseolina*); common rust (*Puccinia sorghi*); common smut (*Ustilago zeae*)
Wheat	760,925,831	49/95	Anthracnose (*Colletotrichum graminicola*); wheat rust (*Puccinia tritici*); *Fusarium* head blight and root rot (multiple *Fusarium* species)
Rice	756,743,722	21/31	Rice blast (*Magnaporthe grisea*); downy mildew (*Sclerophthora macrospora*); root rot (*Fusarium* spp); crown sheet rot (*Gaeumannomyces graminis*)
Oil palm fruit	418,439,313	17/20	Basal stem rot (*Ganoderma boninense*); fruit rot (*Fusarium* spp; *Alternaria* spp; *Aspergillus* spp)
Potato	359,071,403	25/42	Black scurf (*Rhizoctonia solani*); white mold (*Sclerotinia sclerotiorum*); *Fusarium* dry rot (*Fusarium* spp); powdery mildew (*Erysiphe cichoracearum*)
Soybean	353,463,735	38/48	Anthracnose (*Colletotrichum truncatum*); brown stem rot (*Phialophora gregata*); leaf blight (*Cercospora kikuchii*); charcoal rot (*Macrophomina phaseolina*)
Cassava	302,662,494	16/23	Anthracnose (*Glomerella cingulata*); root rot (*Armillaria mellea*); *Fusarium* root rot (*Fusarium oxysporum*); cassava ash (*Oidium manihotis*)
Tomato	186,821,216	25/49	Anthracnose fruit rot (*Colletotrichum* spp.); early blight (*Alternaria alternata*); septoria leaf spot (*Septoria lycopersici*); black mold rot (*Thielaviopsis basicola*)
Barley	157,030,764	36/55	Powdery mildew (*Blumeria graminis*); downy mildew (*Sclerophthora rayssiae*); root rot (*Fusarium graminearum*); leaf rust (*Puccinia hordei*)
Banana	119,833,677	47/56	Panama disease (*Fusarium oxysporum* f. sp. *cubense*); black sigatoka (*Pseudocercospora fijiensis*); black root rot (*Rosellinia bunodes*); anthracnose (*Colletotrichum musae*)
Onion	104,554,458	9/12	Botrytis leaf blight (*Botrytis squamosa*); purple blotch (*Alternaria porri*); neck rot (*Botrytis allii*); onion smut (*Urocystis cepulac*)
Watermelon	101,620,420	7/7	Anthracnose (*Colletotrichum obiculare*); cercospora leaf spot (*Cercospora citrullina*); *Fusarium* wilt (*Fusarium oxysporum* f. sp. *niveum*); powdery mildew (*Podosphaera xanthii*)
Sweet potato	89,487,835	36/45	Leaf mold (*Choanephora cucurbitarum*); gray mold rot (*Botrytis cinerea*); sprout rot (*Rhizoctonia solani*); dry rot (*Diaporthe phaseolorum*)	
Apple	86,442,716	57/83	*Alternaria* rot (*Alternaria alternata*); apple scab (*Venturia inaequalis*); bitter rot (*Colletotrichum gloeosporioides*); core rot (*Pleospora herbarum*)	

Original data were from fao.org and APSNET.org.

A major threat to the global food security is fungal infection of crop plants^34^. Globally, about 8000 fungal pathogens are known to infect crop plants^3^, ^4^, ^35^. Indeed, all our food plants are susceptible to multiple fungal infections. Fungal infections can impact almost all parts of crop plants, from the roots to stem, branches, leaves, flowers, seeds, and fruits. Table 3 summarizes the number of fungal diseases as well as the number of fungal disease agents known to infect the top 15 food plants. Some fungal pathogens such as *Fusarium solani* can infect multiple parts of the same plant such as sweet potatoes, causing different diseases. Similarly, different fungal pathogens can cause the same disease symptom of the same crop. An example is four species in the genus *Fusarium* (*F. zeae, F. graminearum, F. avenaceus*, and *F. culmorum*) as well as *Microdochium nivale* that can all cause scab in wheat. Furthermore, some fungal pathogens can cause diseases in multiple host plants. Species such as *A. alternata, F. oxysporum*, and *Sclerotinia sclerotiorum* can each infect dozens of crop plants and result in significant loss to each crop. For example, *S. sclerotiorum* can cause watery soft rot and other diseases in over 400 species in 75 families of plants^36^.

Throughout human history, fungal diseases have had a significant impact on our food crops at local, regional, national, and/or global levels. Notable examples include the rice blast; anthracnose of corn, wheat, soybean, cassava, and tomato; powdery mildew of potato, tomato, and watermelon; stem rust and *Fusarium* head blight of wheat; root rot of almost all crop plants; chestnut blight; Panama disease of banana; Asian soybean rust; and coffee wilt disease. For example, the global decline in banana yield from 1994 to 1998 was largely due to the widespread Panama disease, caused by the fungal pathogen *Fusarium oxysporum* f. sp*. cubense* tropical race 4, that almost wiped out the Cavendish plantations in Indonesia and Malaysia (Figure 2). This clonal pathogen is now found throughout southeast Asia, China, India, and the Middle East, threatening the global banana industry^35^. Similarly, fungal diseases contributed significantly to decreases in the global wheat yield in 2003, 2005, 2007, 2010, 2012, and 2018 (relative to the respective preceding years), with *F. graminearum* being a major culprit, for example^37^. Even for crops that showed continued growth in global yield, fungal diseases have caused local outbreaks that significantly impacted the local economy and food supply. For example, while the global rice yield increased in 2009 over that in 2008 (Figure 2; 43,416 hg/ha in 2009 vs. 42,919 hg/ha in 2008), the greater Mwea region, a major rice‐growing area in Kenya, experienced 47.9% crop loss as compared to 2008 due to infection caused by the rice blast fungal pathogen *Magnaporthe oryzae* ^38^. Together, it is estimated that each year, over 30% of the global crops are lost due to fungal diseases and that if severe fungal diseases were to happen to all five major crops (maize, rice, potato, soybean, and wheat) all at the same time, over half of the world's population could be starved^3^, ^4^. Several recent reviews summarized the factors contributing to fungal diseases and disease outbreaks in crop fields and orchards^4^, ^34^, ^35^, ^39^.

Plant fungal disease is the result of interaction between the host plant and the fungal pathogen under specific environmental conditions. Thus, factors impacting host plant, fungal pathogen, and/or their interacting environment could all influence the prevalence and severity of each disease. Crop damage in a specific region due to fungal infections may be broadly approximated as follows:

(5) $$D_{F}=\sum_{c=1}^{c=n}\left(P_{D}\cdot N_{D}\right)=\sum_{n=1}^{n=c}\left(N_{C}\cdot F_{C}\cdot N_{F}\cdot F_{F}\cdot N_{D}\right)$$

where *D_F_* represents crop damage due to fungal infection; *P_D_* represents the prevalence of fungal diseases for a specific crop; *N_D_* represents the mean damage of each fungal pathogen to each crop; and *n* is the number of crops under consideration.

The prevalence of fungal disease *P_D_* of each crop in a geographic region may be estimated as a product of the following four terms: *N_C_*, the number of plants of each crop under consideration; *F_C_*, the fraction of plants susceptible to fungal infections; *N_F_*, the total number of plant fungal pathogens within and around crop fields; *F_F_*, the mean fraction of fungal pathogens infecting each crop plant; and *N_D_*, the mean damage of each fungal pathogen to each plant. All five terms can vary widely depending on the geographic regions and crops planted. Based on our current knowledge, all crop plants are known to be susceptible to at least one fungal disease, with most crops susceptible to multiple fungal diseases each (e.g., Table 3). At present, some estimates of individual parameters in isolated crops and crop fields to specific pathogen(s) in specific regions are available. However, such data have rarely been integrated to provide a holistic view of crop damage by multiple pathogens on multiple crops that allow quantitative assessments and predictions. Following Equation (3), changes in any of the above terms could impact the degree of crop damage due to fungal infections, which may be expressed as follows, after time *T*:

(6) $$D_{F+T}=D_{F}\cdot {\left(1+r_{\text{nc}}\right)}^{n}\cdot {\left(1+r_{\text{fc}}\right)}^{n}\cdot {\left(1+r_{\text{nf}}\right)}^{n}\cdot {\left(1+r_{\text{ff}}\right)}^{n}\cdot {\left(1+r_{\text{nd}}\right)}^{n}$$

where *r_nc_* refers to the rate of change in the number of crop species in a defined region per unit time; *r_fc_* refers to the rate of change in the fraction of the considered crop species susceptible to fungal infections in a defined region per unit time; *r_nf_* refers to the change in the total number and virulence of plant fungal pathogens within and around crop fields due to gene flow, de novo mutation, recombination, and/or horizontal gene transfer; *r_ff_* refers to the rate of change in the mean fraction of each crop species being infected by fungal pathogens; *r_nd_* refers to the rate of change in the mean damage of each fungal pathogen to each crop per unit time; and *n* refers to the number of time units under consideration.

At present, quantitative data on the above rates of changes are not available for most geographic regions. However, several host plant factors are known to contribute to their susceptibility to fungal diseases^4^, ^34^, ^35^, ^39^. First, monoculture and the lack of genetic diversity in crop fields can make entire fields and regions highly susceptible to infection by pathogen population(s) with corresponding virulence factor(s). Second, high‐density planting in most agricultural fields facilitates disease transmission among plants. Third, our current crop cultivations rely heavily on chemical fertilizers, the lack of which causes significant stresses in crop plants, making them highly susceptible to fungal infections. Fourth, humans have been increasingly expanding the geographic ranges and ecological niches of crop plants, exposing them to potential new fungal (and other types of microbial) pathogens that are absent in their native ranges.

Similarly, several fungal factors are known to contribute to the fungal infectious diseases in crop plants. These include (i) pathogen species diversity within and around crop fields; (ii) pathogen population size; (iii) pathogen dispersal ability; (iv) pathogen host range; (v) pathogenicity factors including invasiveness, survival, and reproduction within host plants; and (vi) resistance of pathogens to agricultural fungicides^4^, ^34^, ^35^, ^39^. Increases in any of the above could lead to more prevalent and more severe fungal diseases in crop plants. Most plant fungal pathogens have their centers of diversity around the geographic origin of their host plants where the host–pathogen arm race has occurred over millennia and likely reached a dynamic equilibrium. When a host plant expands its geographic range, through either natural dispersal or anthropogenic activities, sooner or later, the pathogen follows to wherever the host plants are expanded. In addition, indigenous fungal pathogens may switch from original native hosts to infect the newly introduced crop plants. During their interactions, new pathogen genotypes may emerge that can cause disease outbreaks through a variety of genetic mechanisms such as mutation, recombination, hybridization, and horizontal gene transfer^35^, ^39^.

Any environmental condition that contributes to increases in the diversity, population size, dispersal, host range, pathogenicity, and fungicide resistance of fungal pathogens as well as to increased host plant susceptibility to fungal infections could result in higher disease prevalence and/or more severe disease symptoms. Following the points raised above, contributing environmental factors that can lead to greater host susceptibilities to fungal infections may include extreme temperatures, excess or limited water availability/humidity, inadequate soil nutrient level, planting the same crop or crop variety consecutively, and trading of infected seedlings and germplasm that may inadvertently facilitate pathogen dispersals. For the fungal pathogens, the presence of infected materials, alternative hosts, permissible temperature, abundant moisture, and nutritional substrates suitable for fungal pathogen growth and reproduction, strong air currents, and anthropogenic activities could all lead to increased pathogen population size and distribution range. Some of these factors have been modeled for predictive purposes of one or few specific components in crop–pathogen interactions, for example^40^, ^41^. However, significant challenges remain^42^. Among these, climate change‐induced frequent and severe weather events such as flooding and drought have been found to play an increasingly important role in weakening a plant's defense system, increasing susceptibilities to fungal infections, and leading to rapid increases in fungal pathogen populations. Indeed, global warming and climate change have led to expanded ranges of fungal plant pathogens and more frequent disease incidences^43^.

Despite the challenges posed by fungal (and other microbial) pathogens and pests, the continued overall increase in yield over the last 60 years for most major crops suggests that overall, we have been managing the fungal (other) threats to crop plants reasonably well. Indeed, new pathogen‐resistant crop cultivars, stricter quarantine measures, and better cultivation and management strategies have been developed and continue to be implemented to ensure sustainable food supply. However, as mentioned above and shown in Figure 2, in many regions of the world, fungal pathogens have caused severe losses to one or few crops and led to food and commodity shortages and economic hardships for those communities whose livelihood is dependent on those crops^35^, ^37^, ^38^. This trend will likely continue. Diversifying crops at the local level to include different cultivars and different crop species will strengthen local resilience against fungal threats. Unfortunately, such a measure alone will unlikely be sufficient. Due to their fast life cycle and large population size, fungal pathogens can evolve very rapidly. In addition, due to their rapid dispersal abilities, border quarantine measures provide no guarantee to prevent pathogen spread among regions and countries. Furthermore, the global climate and soil environments are changing. Thus, it is critical that we develop a holistic understanding of how the various factors impacting host plants, fungal pathogens, and their interacting environments quantitatively contribute to crop losses. Long‐term monitoring of both experimental plots and crop farms for variables, such as host and pathogen distributions, disease prevalence and severity, the effectiveness of fungicide usages, and environmental variables, are needed to identify the key parameters impacting crop losses due to fungal (and other microbial) diseases. The applications of real‐time quantitative polymerase chain reaction markers for pathogen detections, local and regional tracking of spatial and temporal crop field information based on remote sensing and geographic information system, and machine learning to analyze the collected big data should help toward developing better quantitative models for both short‐ and long‐term predictions^35^. Coupled with the development of new fungicides (including biocontrol agents) and new application strategies of existing fungicides (e.g., fungicide combination treatments), the refinements of such models should help develop targeted management plans for specific crops and/or geographic regions.

## THREATS OF MYCOTOXINS IN FOOD

Aside from directly threatening food security by causing diseases on crop plants, fungi can also spoil food and contaminate foods by producing a diversity of mycotoxins either during their growth on crops (with or without influencing crop productivity) or postharvest. Mycotoxins are secondary metabolites that are toxic, carcinogenic, and/or mutagenic and they can be present in high concentrations in food and feed products. Over 300 mycotoxins have been identified and the major ones include aflatoxins, fumonisins, ochratoxin, patulin, trichothecenes, and zearalenone^5^, ^44^. Table 4 summarizes the major mycotoxins, the main fungal agents that produce them, the commonly affected crops and crop products, and their toxic effects. Most mycotoxins in our food and food products are produced by species in a relatively small number of ascomycete genera *Alternaria, Aspergillus, Fusarium*, and *Penicillium*.

**Table 4:** Major mycotoxins, mycotoxin‐producing fungi, and the foods that they commonly affect.

Mycotoxin	Main fungal agents	Commonly affected foods	Toxic effects
Aflatoxins	*Aspergillus flavus, Aspergillus parasiticus, Aspergillus nomius*	Cereals (corn, sorghum, wheat, and rice), oilseeds (soybean, peanut, sunflower, rapeseed, and cotton seeds), spices (chili peppers, black pepper, coriander, turmeric, and ginger), and tree nuts (pistachio, almond, walnut, coconut, and Brazil nut)	Acute liver damage, genotoxicity, and cancer
Ochratoxins	*Aspergillus ochraceus, Penicillium verrucosum, Aspergillus carbonarius*	Cereals (corn, wheat, barley, oats, rice, and sorghum) and cereal products, coffee beans, dry vine fruits, wine and grape juice, spices and liquorice, cocoa, and cheese	Kidney damage and kidney cancer
Fumonisins	*Fusarium verticillioides, Fusarium proliferatum, Alternaria alternata*	Cereals (corn, rice, wheat, and barley), soybean, and their products	Acute toxicity, immunotoxicity, and reproductive toxicity
Zearalenone	*Fusarium graminearum, Fusarium culmorum, Fusarium cerealis, Fusarium equiseti, Fusarium verticillioides, Fusarium incarnatum*	Cereals (corn, wheat, barley, oats, rice, and sorghum) and cereal products	Infertility, abortion, and other reproductive problems
Deoxynivalenol	*Fusarium graminearum, Fusarium culmorum*	Cereals (corn, wheat, oats, barley, rice, and other grains) and cereal products	Acute temporary nausea, vomiting, diarrhea, abdominal pain, headache, dizziness, and fever
Patulin	Many species in genera *Aspergillus* (e.g., *A. clavatonanicus*), *Byssochlamys* (e.g., *B. nivea*), and *Penicillium* (e.g., *P. expansum*)	Apples, apple juice, and concentrate, other fruits, and grains	Genotoxicity, immunotoxicity, edema, and hemorrhage in the brain and lungs; capillary damage in the liver, spleen, and kidney; paralysis of the motor nerves; and convulsions
Gliotoxin	*Aspergillus fumigatus, Gliocladium fimbriatum, Trichoderma* spp.	Fruits and fruit products	Immunosuppression, neurotoxicity, lymphomas, and mammary tumors

At present, the global economic losses, healthcare costs, and human life losses due to mycotoxin contamination of foods are largely unknown. According to the UN FAO, an estimated 25% of the world's crops are contaminated with unacceptable levels of mycotoxins^45^. A range of crop products, from grain crops such as rice, maize, wheat, and barley to peanuts, tree nuts, grapes, and coffee are commonly contaminated by mycotoxins. In the United States alone, contamination by aflatoxins was estimated to cause an annual loss of corn ranging from $52 million to $1.68 billion^46^. Overall, the amount of crop loss postharvest due to mycotoxin contamination may be approximated as follows:

(7) $$L_{\text{MT}}=\sum_{c=1}^{c=n}\left(\text{Nc}\cdot \text{Fc}\;\cdot \;\text{Wc}\cdot \text{Vc}\right)$$

where *L_MT_* represents the total crop loss under consideration due to mycotoxin contamination; *c*_1‐>*n*_ is the number of crops under consideration; *Nc* is the total number of harvested/storage batches for each crop under consideration; *Fc* is the fraction of harvested/stored batches for each crop contaminated by mycotoxins; *Wc* is the weight of each batch; and *Vc* is the value per unit weight of each crop. Different from other fungal threats, crop losses due to mycotoxin contamination at the national levels are often tracked for major crops such as maize and others listed in Table 4. For example, a recent report of 892 maize samples in the Kingdom of Eswatini from 2001 to 2021 showed that several aflatoxins (aflatoxin B1, B2, G1, and G2) and Zearalenone were frequently detected in both maize grain and processed maize products throughout the 20 years, with a significant number of samples (38/892, 4.3%) containing more than two or more mycotoxins^47^. Similarly, about 50% of the 2018 harvested maize in Eastern African community countries was reported to be contaminated by mycotoxins, with a significant proportion exceeding the regulatory thresholds^48^. However, these national surveys of mycotoxin contaminations are likely underestimates of the true levels. For example, most heavily contaminated and spoiled crop products are typically discarded on site by farmers and are not sold to distributors where mycotoxin testing is typically conducted.

Significant progress has been made in understanding mycotoxin production by fungi and their mechanisms of toxicity on host cell lines, tissues, and organs^5^, ^44^. The increased understanding has led to the development of a variety of new mycotoxin‐monitoring and food processing, transportation, and storage techniques. However, mycotoxin contamination persists. The persistence of mycotoxins in food and feedstuff is largely due to the ubiquitous distributions of mycotoxin‐producing fungi. Almost all mycotoxin‐producing fungi can grow on a diversity of crops, crop residues, and foods in a variety of environmental conditions, including in foods served in hospitals where hosts with compromised health conditions are commonly found^5^, ^49^. With climate change causing more frequent precipitation and flooding^50^, there will likely be more favorable conditions for the growth of mycotoxin‐producing fungi and more mycotoxins in our environments. For example, the production of Zearalenone increases with increasing temperature when air humidity is at or above 20%, a humidity commonly found in most environments^51^. Consequently, climate change will likely make it more difficult and expensive to control these fungi and their mycotoxin production in many parts of the world. Compounding the threat by mycotoxins is that many of the mycotoxin‐producing fungi such as species in genera *Alternaria, Aspergillus*, and *Fusarium* also contain a diversity of allergens (Table 2).

## FUNGAL DISEASES IN HUMANS

Among all the fungal threats, the most direct and perhaps the most familiar to us are those causing human infections. Indeed, infectious diseases have played a major role in shaping the history of humans. The ongoing COVID‐19 pandemic is but one of many pandemics through the ages. The major pandemics most familiar to us are those caused by bacteria (e.g., the biblical pharaonic plagues in Ancient Egypt around 1715 BC, the “cocoliztli” epidemics in Mexico during the 16th century, and the Black Death bubonic plague in Europe in the mid‐14th century) and viruses (e.g., the pandemic influenza virus that swept through the world in 1918–1919 and the ongoing global AIDS epidemic since the early 1980s). However, though less sensational, fungal diseases in humans are similarly widespread and typically more difficult to treat than both bacterial and viral diseases. For example, superficial mycoses, such as dermatophyte infections of the skin and nails, affect more than 25% of the population worldwide during their lifetime, with chronic fungal nail infection alone affecting over 400 million people each year^52^. Based on data in the Leading International Fungal Education portal from 2013 to 2017 that included selected hospitals/clinical microbiology labs in 43 countries on the prevalence of selected fungal diseases from each country, it was estimated that fungal pathogens were directly responsible for about 1.5–2 million deaths annually and compromised the life of over 2 billion people worldwide^53^ (Figure 1 and Table 5). Among the chronic and/or invasive infections annually at the global level, there are ~3,000,000 cases of chronic pulmonary aspergillosis, ~1,000,000 cases of fungal keratitis, ~700,000 cases of invasive candidiasis, ~500,000 cases of *Pneumocystis jirovecii* pneumonia, ~250,000 cases each of cryptococcal meningitis and bloodstream aspergillosis, and ~100,000 cases of disseminated histoplasmosis^53^ (Table 5). The direct annual medical cost of fungal diseases is estimated at >7.2 billion dollars in the United States alone^54^. The major human fungal infection burdens, including the major agents, are summarized in Table 5.

**Table 5:** Estimated global burden of human fungal infections.

Fungal infection	Annual incidence	Global burden^a^	Main agents
Superficial infection			
Skin, hair, nail		~1,000,000,000	*Trichophyton (T. rubrum; T. mentagrophytes; T. tonsurans; T. violaceum; T. schoenleinii; T. soudanense); Microsporum (M. canis; M. audouinii; M. rivalieri; M. ferrugineum); Epidermophyton floccosum; Candida (C. albicans; C. parapsilosis; C. glabrata); Lacazia loboi*
Fungal keratitis		~1,000,000	*Candida* (e.g., *C. albicans; C. glabrata; C. tropicalis); Fusarium* (e.g., *F. avenascus; F. oxysporum; F. solani); Aspergillus* (e.g., *A. fumigatus; A. flavus*)
Mucosal infection			
Oral candidiasis	~2,000,000		*C. albicans; Candida (Torulopsis) glabrata; Candida parapsilosis* complex; *Candida tropicalis; Candida krusei; Candida auris; Candida (Meyerozyma) guillermondii; Candida rugosa*
Esophageal candidiasis	~1,300,000		
Recurrent vulvovaginal candidiasis		~134,000,000	
Chronic/invasive/multiple organ infections			
Blastomycosis		~3000	*Blastomyces dermatitidis; Blastomyces gilchristii*
Chromoblastomycosis		>10,000	*Fonsecaea pedrosoi; Phialophora verrucosa; Cladophialophora carrionii*
Chronic pulmonary aspergillosis		~3,000,000	*Aspergillus fumigatus*
Coccidioidomycosis		~25,000	*Coccidioides immitis; Coccidioides posadasii*
Cryptococcosis	~250,000		*Cryptococcus neoformans* complex; *Cryptococcus gatti* complex
Emergomycosis		>100 (based on case reports)	*Emergomyces africanus; Emergomyces canadensis; Emergomyces orientalis; Emergomyces europaeus; Emergomyces pasteurianus*
Histoplasmosis	~500,000		*Histoplasma capsulatum* complex
Invasive aspergillosis	>300,000		*Aspergillus fumigatus*
Invasive candidiasis	~750,000		*C. albicans; C. glabrata; C. tropicalis; C. auris*
Mucormycosis		~200,000	*Rhizopus oryzae; Mucor indicus*
Mycetoma		~9000	*Madurella mycetomatis*
Paracoccidioidmycosis		~4000	*Paracoccidioides brasiliensis; Paracoccidioides lutzii*
Pneumocystis jirovecii pneumonia	~500,000		*Pneumocystis jirovecii*
Sporotrichosis	>40,000		*Sporothrix schenckii; Sporothrix brasiliensis; Sporothrix globose; Sporothrix luriei*
Talaromycosis	~17,300		*Talaromyces (*formerly *Penicillium) marneffei*

The estimated annual incidence and global burden numbers were mainly from Table 1 in Bongomin et al.^53^ Other data sources include who.int (accessed June 22, 2022) and Kohler and colleagues^2^, ^52^, ^53^, ^54^, ^55^.

^a^ Global burden estimates were based on combined data that encompass annual incidence, period, or total prevalence, and in the case of recurrent vulvovaginal candidiasis, annual prevalence, from different countries/regions.

At present, about 200 fungal species are known to be associated with human diseases. Most of these human fungal pathogens have extensive environmental reservoirs and can complete their reproduction outside of human hosts. For many species such as those in genera *Candida* and *Aspergillus*, they are ubiquitously found in our living environments. Most of us are exposed to them daily through the food that we eat, the liquids/juices that we drink, and the air that we breathe. However, for most immunocompetent hosts, when the exposed pathogen dose is low, most fungal agents that we encounter can be effectively cleared/controlled and there may not be any noticeable disease symptom. In contrast, for immunocompromised hosts, exposure to even a small dose of fungal pathogen can result in severe disease symptoms, including death. Indeed, like those of other host–pathogen interactions, human fungal disease is the result of interaction between individual hosts and fungal pathogens under specific environmental conditions. Therefore, factors related to host susceptibility, fungal pathogen load and virulence, and their interacting environment could all influence the outcome of each exposure to a fungal pathogen. The number of fungal infectious disease incidences in a population at a specific time unit may be approximated as:

(8) $$N_{\text{FI}}=\sum_{f=1}^{f=m}\left(N_{T}\cdot F_{S}\cdot F_{F}\right)$$

where *N_FI_* represents the total number of fungal infectious disease incidences in a population at a specific time unit (e.g., a year); *m* is the number of human fungal pathogens in the community; *N_T_* is the total number of people in the community; *F_S_* is the fraction of the population susceptible to infection by each specific fungal pathogen; and *F_F_* is the fraction of each fungal pathogen reaching the infectious dose and causing disease symptoms in the susceptible hosts. *F_F_* could be further divided into different categories based on disease types and disease severities. How fungal diseases are resolved will depend on host immune response and the effectiveness of medical interventions through diagnosis and antifungal treatments.

(9) $$N_{\text{FI}-R}=\sum_{f=1}^{f=m}\left(N_{T}\cdot F_{S}\cdot F_{F}\cdot F_{R}\right)$$

where *N_FI__− R_* refers to the remaining incidences of fungal infection after treatment (i.e., the initial infections have become recurrent or persistent infections or even death); *F_R_* refers to the fraction of diseased patients who failed to mount adequate immune response and/or failed subsequent medical intervention (e.g., due to antifungal drug resistance).

All five terms (*m*, *N*_*T*,_ *F_S_*, *F_F_*, and *F_R_*) can vary widely depending on the populations under consideration. Based on our current knowledge, at the population level, all human populations, regardless of their age, sex, and geographic origin, are known to be susceptible to at least one fungal disease. However, changes in any of the five terms could impact the incidences of fungal infectious diseases in a community, which may be approximated as follows, after time *T*:

(10) $$N_{\text{FI}+T}=N_{\text{FI}}\cdot {\left(1+r_{\text{nt}}\right)}^{n}\cdot {\left(1+r_{\text{fs}}\right)}^{n}\cdot {\left(1+r_{\text{ff}}\right)}^{n}$$

where *r_nt_* refers to the rate of change in the number of people in a defined population per unit time; *r_fs_* refers to the rate of change in the fraction of people susceptible to fungal infections in a defined region per unit time; *r_ff_* is the rate of change in the fraction of each fungal pathogen reaching the infectious dose and causing disease symptoms in the susceptible hosts per unit time; and *n* is the number of time units under consideration. Similarly, after time *T*, how fungal diseases are resolved in a population will depend on changes in host response and/or in advances of medical intervention (*r_fr_*).

(11) $$N_{\left(\text{FI}+T\right)-R}=N_{\text{FI}+T}{\left(1+r_{\text{fr}}\right)}^{n}$$

Many models have been developed to study human infectious disease epidemiology^54^, ^55^. Most studies have focused on viral and bacterial diseases and how they spread and persist in human populations. For fungal disease epidemiological studies, previous studies have shown that a number of factors related to human hosts, fungal pathogens, and their interacting environments contribute to the prevalence and severity of fungal diseases in humans^52^, ^53^, ^54^, ^55^, ^56^, ^57^, ^58^. Briefly, human host factors contributing to fungal infectious diseases include (i) infection by the human immunodeficiency virus (HIV); (ii) the application of broad‐spectrum antibiotics to treat bacterial infections; (iii) the application of immunosuppressive drugs for organ transplantation and other diseases; (iv) chemotherapy for cancer; (v) diabetes; (vi) old age; and (vii) other host‐debilitating conditions. The fungal factors that can impact fungal infectious diseases in humans include (i) pathogen species diversity within and around our living environments; (ii) pathogen population size around humans; (iii) pathogen dispersal ability; (iv) pathogenicity factors such as those associated with invasiveness, survival, and reproduction within hosts; and (v) susceptibility to antifungal drugs. Any environmental factors that contribute to increases in the diversity, population size, dispersal, pathogenicity, and drug resistance of fungal pathogens and/or to increases in host exposure and susceptibility to fungal pathogens could all lead to an increase in fungal disease prevalence and severity in humans. The reverse is also true.

Globally, our ability to manage HIV infection has increased significantly and this has contributed to declining opportunistic fungal infections among AIDS patients in most of the developed countries. However, with an aging global population (Figure S1) and predicted increases in the number of people (i) needing organ transplantation, (ii) undergoing chemotherapy due to various forms of cancer, (iii) becoming severely diabetic, and (iv) suffering from other debilitating conditions, there will likely be an overall increase in the number (and increasing proportion) of people susceptible to fungal diseases. The changes will be different in geographic regions. For example, due to the increasing prevalence of HIV infection in sub‐Sahara Africa and the limited access to antiviral therapy, opportunistic fungal infections will likely persist across most demographic groups in that part of the world. In contrast, in developed countries, fungal infections in non‐HIV‐infected people will be more common due to aging populations and other immunocompromising conditions.

In terms of fungal pathogens, several broad patterns are also emerging. First, anthropogenic activities are accelerating pathogen movements both within and across regions^59^, ^60^, ^61^, ^62^, ^63^. Such gene flow increases local pathogen diversity while homogenizing the pathogen populations across regions^59^, ^60^, ^61^, ^62^, ^63^, ^64^, ^65^. Pathogen movements also create opportunities for mating and hybridization among previously segregated populations, which can lead to the generation of new and more virulent pathogens, for example^66^. Second, similar to that described in earlier sections for other fungal threats, increases in both temperature and moisture due to climate change at both local and global levels have contributed and will likely continue to contribute to increases in the population size of human fungal pathogens^67^. Third, intrinsically drug‐resistant species and drug‐resistant genotypes in traditionally drug‐sensitive species are becoming increasingly frequent both in humans and in the surrounding environments^2^, ^59^, ^64^. At least some of the increase has been driven by our heavy reliance on agricultural fungicides to maintain crop productivity. Some of the agricultural fungicides have similar mechanisms of action as medical antifungal drugs. The applications of such fungicides would select for antifungal resistance and facilitate the spread of drug‐resistant genes among human fungal pathogens in diverse ecological niches in many parts of the world, for example^64^, ^65^, ^68^, ^69^, ^70^.

The currently known fungal species associated with human diseases belong to diverse phylogenetic groups within three phyla: *Zygomycota*, *Ascomycota*, and *Basidiomycota*. This distribution pattern is consistent with multiple independent origins of fungal pathogenicity in humans. However, human fungal pathogens are not randomly distributed across these three taxonomic groups and the types of diseases that they cause can differ among different groups of fungi and among geographic regions. For example, 144 species from 92 genera have been reported to cause fungal keratitis, with different geographic regions showing differences in their species distributions^71^. Similarly, ringworm is mainly caused by about 40 species in three genera: *Trichophyton, Microsporum*, and *Epidermophyton* ^72^; fungal meningitis is mainly caused by two *Cryptococcus* species complexes (or eight described *Cryptococcus* species)^73^; and mucosal and bloodstream yeast infections are mainly caused by *Candida* species, where 42 species in this genus are known to be pathogenic to humans, with geographic regions showing different genera and/or species distributions^2^, ^74^, ^75^, ^76^. In addition, at the global level, within individual taxonomic groups, the frequencies of individual species causing various forms of infection are not evenly distributed. For example, among the 42 *Candida* species, five (*C. albicans, C. glabrata, C. tropicalis, C. parapsilosis*, and *C. krusei*) are responsible for over 90% of candidiasis cases^2^, ^74^. Similarly, *Cryptococcus neoformans* (serotype A) is responsible for over 80% of the global cryptococcosis^2^, ^73^.

Epidemiological surveys have identified several notable features in the geographic distributions of both fungal diseases and fungal pathogens. At the disease level, differences in host demographics and the economic and healthcare status among countries and regions are reflected in fungal disease burdens and in the overall health of populations. For example, emergomycosis, mucomycosis, mycetoma, and chromoblastomycosis are more commonly found in developing regions such as South and Southeast Asia and sub‐Sahara Africa^53^, ^56^. Similarly, fungal pathogen populations are not evenly distributed. For example, pathogens (Table 5) responsible for the diseases talaromycosis, paracoccidioidmycosis, coccidioidomycosis, blastomycosis, and histoplasmosis are mainly found in Southeast Asia, South America, the southwestern part of North America, and around the Great Lakes in North America, respectively^2^, ^53^. Even for globally prevalent diseases such as Cryptococcosis, the causal agents can show geographic specificity in their genotype distributions^73^. However, for many fungal pathogens in the genera *Aspergillus* and *Candida*, there is abundant evidence for recent gene flow among geographic populations, largely caused by human movements and anthropogenic activities, for example^59^, ^60^, ^63^. Consequently, the distinctiveness and genetic differentiations among geographic populations of human fungal pathogens are becoming increasingly blurred. Together, an increasing pathogen diversity is expected at the local level, while across geographic populations, there will be decreased differences at both the species level and the population level within individual species.

For several reasons, the documented number of fungal species capable of causing human diseases has been continuously changing. First, there is a diversity of fungal species concepts and the fungal species delineation criteria have been evolving^77^. Using molecular markers such as multiple gene genealogical analyses and new species delineating criteria, many previously recognized individual species have been found to contain multiple species within each. Examples of these include *Candida parapsilosis, Candida haemulonii, Cryptococcus neoformans*, and *Cryptococcus gattii* ^73^, ^75^, ^76^, ^78^. Second, while many fungi have been found to be associated with disease symptoms, the causal relationships between these fungi and the observed disease symptoms remain to be determined for a significant proportion of them. This is especially true for fungi isolated from the surfaces of the skin, mucus membrane, and the hair and nails. Third, new human fungal pathogens are continuously emerging. Examples include (i) *Cladophialophora bantiana*, which can cause brain infection in healthy hosts without any known history or portal of entry^79^, (ii) *Candida auris*, which was first reported in 2009 but has since caused nosocomial outbreaks in over 50 countries^80^, ^81^ (Figure 1), and (iii) *Exserohilum rostratum*, which led to the death of over 60 people in the United States in the mid‐2010s due to contamination of steroids^82^. Indeed, *C. bantiana* and *C. auris* were recently considered 2 of the 10 most feared fungi by a group of mycology experts^83^.

Direct fungal threats to human health will persist and most likely increase in certain populations. For example, the current COVID‐19 pandemic has exacerbated many fungal infections, including pulmonary aspergillosis, mucormycosis, candidiasis, and several endemic mycoses^84^. While estimates of the global fungal disease burdens are becoming increasingly robust, accurate data from >100 countries remain unavailable for most fungal diseases. Given the increasing digitization of healthcare records across the world, it should be possible to have all fungal disease incidences recorded, deposited, and summarized in a timely manner. Such data will help guide the rapid deployment of appropriate resources to prevent and control fungal diseases. Indeed, our increased understanding of both host factors and pathogen factors that contribute to fungal infectious diseases is generating significant advances to help tackle these fungal threats. The advances include developing (i) fast and effective diagnosis tools to detect fungal infections so that early targeted treatments can be quickly implemented; (ii) programs for effective use of current antifungal drugs and agricultural fungicides to maximize treatment effects while minimizing drug resistance in both clinics and agricultural fields; (iii) new antifungal drugs or drug combinations; (iv) vaccines against the major fungal pathogens; and (v) better logistics in delivering effective approaches across the globe, for example^53^, ^85^, ^86^. While threats of fungal infections in humans will not go away, if timely diagnosis and treatments are prescribed, it has been estimated that >80% of deaths caused by fungal diseases could potentially be prevented^53^.

## CONCLUSIONS AND PERSPECTIVES

Here, I review and assess the major fungal threats to humans, including the diversity of factors influencing each threat. Among the many factors, several have emerged to contribute to multiple fungal threats, including climate change, anthropogenic activities, fungal mutation and evolution, and human demographics. Though not discussed here, fungal threats to the environment and wildlife can also directly or indirectly impact humans^87^. For example, the aquatic chytrid fungi *Batrachochytrium dendrobatidis* and *Batrachochytrium salamandrivorans* cause global chytridiomycosis in amphibians, and the ascomycete fungus *Pseudogymnoascus destructans* causes white‐nose syndrome in hibernating bats in North America. These fungal pathogens have devasted the global amphibian and the North American bat populations and the fungal populations are rapidly evolving, with anthropogenic factors playing significant roles in their population dynamics^1^, ^88^. Amphibians and bats play important roles in agriculture and human health by influencing the dynamics of insect populations that may act as pollinators, agricultural pests, and vectors of human infectious disease agents.

It is often argued that a major goal of scientific research is to identify the smallest number of variables that can explain the broadest set of phenomena. In threat analyses, identifying the key variables can provide critical information for how to minimize threats. While there is an abundance of literature on microbial threats, most studies have focused on the short‐term dynamics of disease transmissions, especially on viral and bacterial diseases in human populations. However, fungal populations differ from viral and bacterial populations in several fundamental ways, including their relatively close evolutionary relationships with animals, and for many fungal threats of humans, the presence of extensive environmental reservoirs, the potential to undergo both sexual and asexual reproduction, and the ability to produce abundant spores that can be easily dispersed by air currents and remain dormant for an extended period. Such fungal features require approaches different from those used for viruses and bacteria to analyze and to quantify their impacts. Here, I provide extremely simplified approximations to quantify various fungal threats to humans and review and discuss the diversity of factors that impact each threat. At present, much of the fungal threat research is descriptive and there are limited quantitative data to generate predictions and design response strategies. Concerted medium‐ to long‐term monitoring and surveillances are needed to obtain accurate quantitative information on all known and currently unknown but potentially important contributors for each fungal threat.

## Supporting information

Supplementary Figure 1.
