# Supplementary material for: Assessing global fungal threats to humans
Source: mLife. 2022 Sep 22;1(3):223–40. doi: 10.1002/mlf2.12036 (PMC10989982; doi:10.1002/mlf2.12036)

Supplementary Figure 1. Observed and predicted global human population size and demographic structure from 1950 to 2100. Figure adapted with permission from ref. 59, under a Creative Commons license CC By 4.0.

Supplementary Figure 1


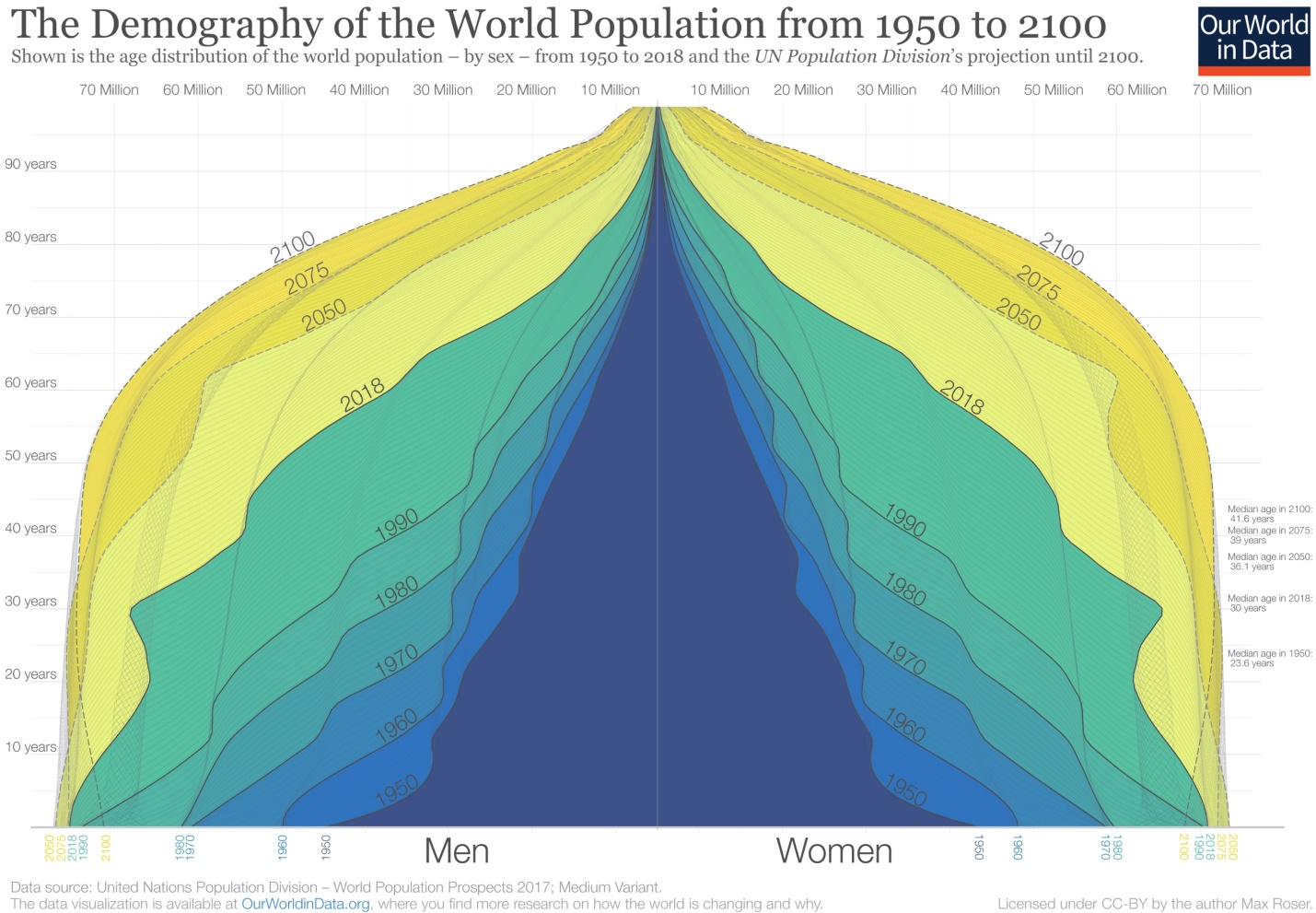

Supplement: Supplementary file 1 — Supplementary Figure 1. [file MLF2-1-223-s001.docx]
